# The role of cell death pathways in respiratory viral infection and vaccination: two sides of the same coin

**DOI:** 10.3389/fimmu.2025.1650960

**Published:** 2025-09-05

**Authors:** Sun Min Lee, Eui Ho Kim

**Affiliations:** ^1^ Viral Immunology Laboratory, Institut Pasteur Korea, Seongnam, Republic of Korea; ^2^ Department of Advanced Drug Discovery & Development, University of Science and Technology (UST), Daejeon, Republic of Korea

**Keywords:** cell death pathways, damage-associated molecular patterns (DAMPs), viral infections, vaccines, adjuvants

## Abstract

Cell death pathways play contrasting roles in physiological processes such as responses to viral infections and vaccinations, potentially exerting either detrimental or beneficial effects. On one hand, uncontrolled cell death accompanied by the release of damage-associated molecular patterns (DAMPs) can lead to excessive inflammation and tissue damage. On the other hand, when properly regulated, these processes help establish an immunocompetent environment by activating innate immunity, which in turn stimulate antiviral immune responses. These mechanisms have emerged as promising targets for the development of effective antiviral therapeutics, immunotherapies, and vaccines. Recent advances have elucidated key aspects of cell death and DAMP pathways, highlighting their association with upstream viral sensors, their capacity to regulate immune responses, and their potential as therapeutic targets in the context of respiratory viral infections such as influenza virus and SARS-CoV-2. In this review, we discuss the advantages and disadvantages of cell death and DAMP pathways, focusing on their roles in antiviral immunity and pathogenesis of respiratory viral infections, and vaccine immunogenicity.

## 1 Introduction

Cell death is a physiological process essential for development and homeostasis but can also result from cellular stress or tissue damage. Regulated cell death (RCD) refers to cell death driven by signal transduction pathways, including physiological ‘programmed cell death’ and stress-induced forms. RCD encompasses apoptosis, pyroptosis, necroptosis, and ferroptosis, each controlled by distinct molecular pathways, unlike unregulated accidental necrosis (1). Apoptosis, the first defined RCD, is non-inflammatory and triggered by intrinsic or extrinsic signals (death receptor ligation), activating initiator (caspase-8/9) and effector (caspase-3/7) caspases. Apoptotic cells form membrane blebs and apoptotic bodies, which partition cellular contents within the membrane. This controlled packaging facilitates efficient clearance by phagocytes without triggering inflammation (2). Pyroptosis is a necrotic RCD driven by inflammasome activation and gasdermin (GSDM) cleavage (1), leading to membrane rupture and release of cellular contents. Microbe-associated molecular patterns (MAMPs, e.g., toxins, flagellin, and double-stranded RNA), and damage-associated molecular patterns (DAMPs, e.g., ATP, nucleic acids, and uric acid) can interact with canonical inflammasome sensors—NOD-like receptor (NLR) family pyrin domain-containing 1 (NLRP1), NLRP3, absent in melanoma 2 (AIM2), NLR family CARD domain-containing 4 (NLRC4), and Pyrin—leading to the activation of caspase-1 (or caspase-11/4/5 in the non-canonical pathway), and subsequent release of interleukin-1β (IL-1β) and IL-18 (3). Necroptosis is another necrotic form of RCD that is inherently inflammatory. Death receptor signaling from tumor necrosis factor receptor 1 (TNFR1), Fas, death receptor 4 (DR4), and DR5 under apoptosis-suppressive conditions (lacking caspase-8 function) facilitates necroptosis through the activation of the RIPK1–RIPK3–MLKL axis, in contrast to pyroptosis (4, 5). Activated RIPK1 promotes the oligomerization and activation of RIPK3, which subsequently phosphorylates the pseudokinase MLKL (6). Oligomerized MLKL translocates to the plasma membrane, inducing membrane rupture and cell lysis (7). Beyond death receptors, pattern recognition receptors (PRRs) such as Toll-like receptor 3 (TLR3), TLR4, and Z-DNA-binding protein 1 (ZBP1) can also promote necroptosis (8, 9). Additionally, metabolic stress conditions (e.g., glucose deprivation or hypoxia) have been reported to stimulate necroptosis (10, 11). Ferroptosis is a distinct form of necrotic RCD that occurs through iron-dependent membrane lipid peroxidation (12). It is initiated by a reaction between intracellular iron and hydrogen peroxide produced by mitochondria and is executed through downstream processes, including ion fluxes, disruption of ionic homeostasis, and eventual plasma membrane rupture. The susceptibility to ferroptosis depends on the cells’ feature of accumulating lipid peroxide in the membrane, which is up to the amount of intracellular iron and oxidizable lipids. Important regulatory factors also include glutathione (GSH)–glutathione peroxidase 4 (GPX4) pathway and NADPH–ferroptosis suppressor protein 1 (FSP1) systems (13). Though ferroptosis can have beneficial roles in tumor suppression, it also contributes to pathological conditions like organ injury and infections (14).

Cellular factors such as PRRs, caspases, and GSDMs modulate membrane-disruptive activity and regulate the transition from apoptotic to lytic forms of cell death (15). Activation of apoptotic caspases can either suppress necrotic cell death to preserve the apoptotic phenotype or promote its progression into secondary necrosis. For example, caspase-3 and -7 can suppress pyroptosis by cleaving GSDMD at a site distinct from that targeted by caspase-1, which generates the pore-forming N-terminal fragment of GSDMD (16). On the other hand, during a single-stranded RNA virus infection, caspase-3 activation in apoptotic cells expressing the GSDMD-related protein DFNA5 can cleave DFNA5, to induce secondary necrosis or pyroptosis (17). In addition, modification of MLKL and GSDMs also determines their necrotic effector functions. Notably, phosphorylation of GSDME and GSDMA inhibits pyroptosis, while phosphorylation of MLKL at serine 83 suppresses necroptosis (15). The interconnected regulatory mechanisms, downstream of PRRs that detect external threats, allow cells to fine-tune RCD pathways. Through their individual or combined activation during homeostasis, infection, vaccination, or metabolic stress, these pathways can trigger inflammatory and immunogenic responses that are sometimes beneficial and sometimes detrimental—like two sides of the same coin.

While cell death was once viewed as separate from immunity, recent findings show that certain RCD forms and associated DAMPs can initiate inflammation and adaptive immune responses (18–24). Consequently, these pathways are receiving increasing attention for their roles in both health and disease (25). Therefore, their beneficial and detrimental effects on disease progression and medical applications must be thoroughly investigated. This review highlights the immunostimulatory effects of cell death and DAMP pathways, which can exert opposing roles, particularly in respiratory viral infections and vaccine responses.

## 2 Cell death and DAMP pathways in viral infection

Human respiratory viral infections, including those caused by influenza A virus (IAV) and severe acute respiratory syndrome coronavirus 2 (SARS-CoV-2), pose major public health risks with pandemic potential. Upon infection, host cells detect viral RNA via PRRs such as TLRs, RIG-I-like receptors, cGAS–STING, ZBP-1, and NLRs (26). TLRs and RLRs are long known for recognizing viral components and initiating antiviral immune responses. Specifically, double-stranded RNA stimulates TLR3 or RIG-I–MDA5, and single-stranded RNA stimulates RIG-I or TLR7/8 signaling pathway. These interactions induce antiviral signaling, particularly type I interferon (IFN) responses, via their respective pathways (27). Although cGAS canonically senses double-stranded DNA or DNA: RNA hybrids to activate the STING pathway and downstream type I IFN responses, it also plays critical roles in RNA virus infections, including IAV and SARS-CoV-2 (28). For instance, the cGAS–STING pathway has been shown to contribute to type I IFN–mediated immunopathology in the skin, lungs, and endothelium of COVID-19 patients, including endothelial cell death (29). Additionally, IAV has been found to activate the STING pathway in a cGAS-independent manner (30). In contrast to these antiviral PRRs, ZBP-1 and the NLRP3 inflammasome more directly promote cell death pathways. ZBP1 is a key innate sensor of IAV and SARS-CoV-2, initiating inflammatory and cell death pathways (31). Upon infection, ZBP1 recognized viral RNA or viral proteins, to activate RCD pathways (8, 32). The following section will explore detrimental and beneficial roles of cell death-mediated responses that occur primarily downstream of viral recognition by these innate sensors.

### 2.1 Harmful effects in virus-induced pathogenesis

Virus-induced cell death contributes to both host defense and pathogenesis, triggering immune activation while also causing harmful inflammation and tissue damage. The latter effect can exacerbate infection and disease progression. Over the past decade, our understanding of cell death and DAMP pathways in viral disease has advanced significantly.

**Figure 1:**
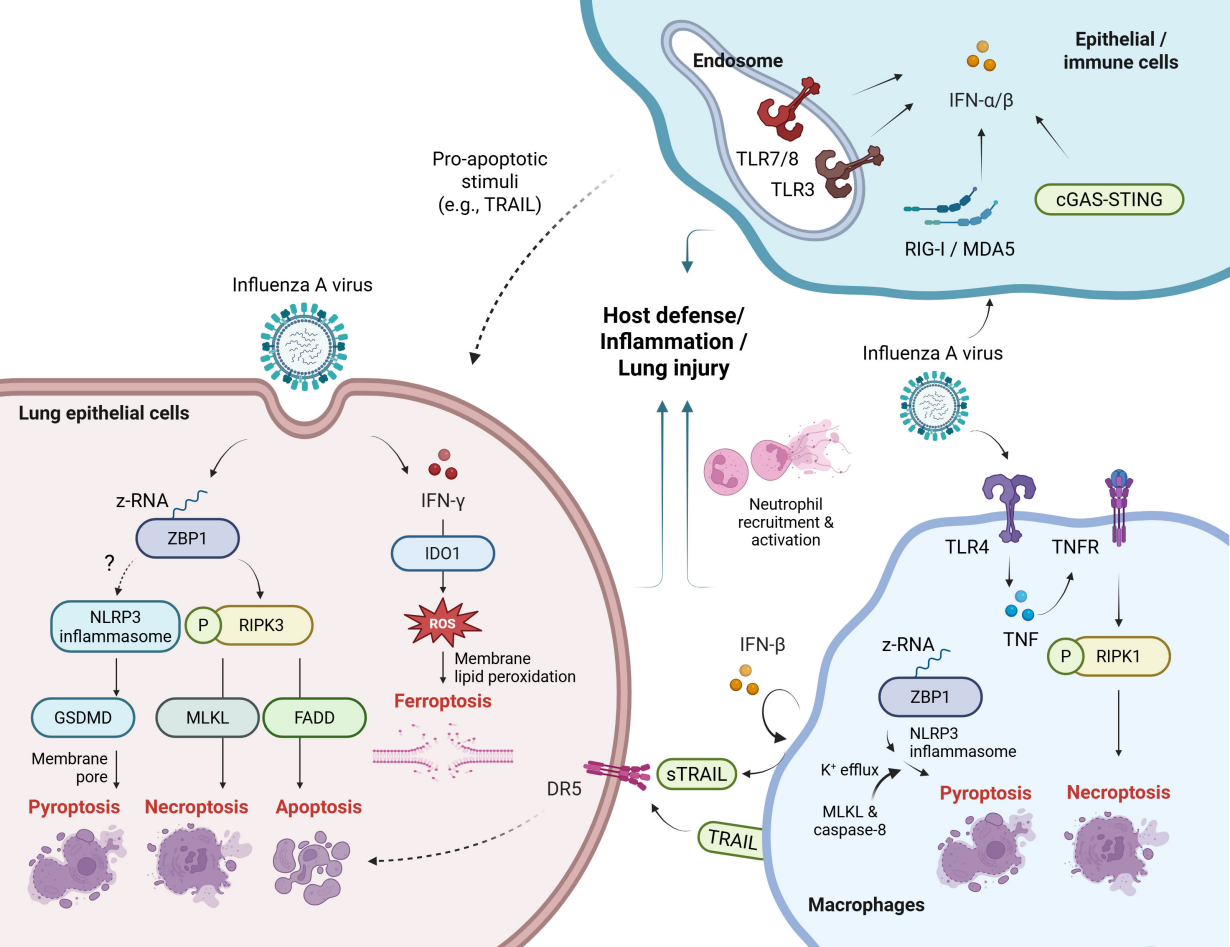
Innate viral sensing and cell death pathways in influenza infection. (Left) In lung epithelial cells, ZBP1 may detect IAV-derived Z-RNA and activates the NLRP3 inflammasome, leading to GSDMD-mediated pyroptosis. ZBP1 also triggers RIPK3-dependent necroptosis via MLKL or apoptosis via FADD. IFN-γ induces IDO1 expression, promoting ferroptosis. These pathways contribute to immune activation or lung injury. (Right) In macrophages, IAV can activate RIPK1-dependent lytic cell death via TLR4 and TNF signaling. ZBP1–NLRP3-driven pyroptosis involves MLKL-mediated K^50^ efflux and caspase-8–mediated inflammation. PRRs sense viral components and induce type I IFNs, among which IFN-β may promote epithelial cell apoptosis via TRAIL–DR5 signaling. (Upper right) Type I IFN responses initiated by PRRs can elicit antiviral immunity or, under certain conditions, facilitate epithelial cell apoptosis. Dotted lines indicate pathways that lack direct evidence or involve multiple undefined mechanisms. sTRAIL, soluble TRAIL. Created with BioRender.com.

As described above, multiple PRRs recognize viral infection and induce type I IFN responses. Besides its well-known antiviral activity, type I IFNs can contribute to pathogenesis by promoting alveolar epithelial cell apoptosis and inflammation (27, 33–35) (**Figure 1**). Briefly, the type I IFNs could sensitize fibroblasts to apoptosis induced by IAV in a FADD/caspase-8-dependent manner (35). Moreover, IFN-β produced by alveolar macrophages have been shown to induce the expression of the pro-apoptotic factor TNF-related apoptosis-inducing ligand (TRAIL) in an autocrine manner, thereby promoting epithelial cell apoptosis and lung injury (33). Type I IFNs derived from plasmacytoid dendritic cells (pDCs), PDCA^+^ NK cells, and PDCA^+^ inflammatory monocytes have also been proposed to contribute to TRAIL–DR5–mediated epithelial cell apoptosis, although TRAIL expression may be differentially induced depending on the cell type (34). More recent evidence for the role of cell death in viral pathogenesis includes findings that Zbp1 deficiency protected mice from IAV-induced mortality by limiting NLRP3-driven inflammation and RIPK3-mediated necroptosis and apoptosis, thereby reducing epithelial damage. Necroptosis promoted pathogenic neutrophil recruitment, increasing lethality (8). The ZBP1 also interacted with the IAV nucleoprotein and polymerase subunit PB1 to trigger pyroptosis, apoptosis, necroptosis, and inflammatory responses through activation of NLRP3 inflammasome and the RIPK1–RIPK3–caspase-8 axis in macrophages (32). Additionally, GSDMD, a pore-forming effector protein, exacerbated inflammation, lung pathology and morbidity during IAV infection. Notably, GSDMD deficiency reduced neutrophil activation and neutrophil extracellular trap (NET)-associated DNA release, underscoring its role in neutrophil-driven inflammation (36). In addition, IAV, or its hemagglutinin (HA) protein, can trigger necroptosis specifically in macrophages via TLR4 and TNF signaling pathways (**Figure 1**). Evidence of lytic cell death included elevated lactate dehydrogenase (LDH) activity and Annexin V/PI (propidium iodide) staining. Necroptosis was confirmed by detecting phosphorylated RIPK1 (Ser166) in human and murine macrophages and by inhibition with Necrostatin-1. Importantly, the clinically approved TNF inhibitor etanercept reduced macrophage necroptosis and cytokine storm in IAV-infected mice (37). This represents a significant advancement over previous studies that had only proposed the involvement of the TLR3/4–TRIF pathway in IAV-induced necroptosis, without providing direct mechanistic evidence (38).

Beyond each individual cell death pathway, recent studies show that necroptosis and apoptosis pathway factors can functionally converge to promote pyroptosis and inflammasome activation during viral infections such as IAV. For example, MLKL promotes NLRP3 inflammasome activation via potassium (K^+^) efflux in macrophages. Furthermore, combined depletion of MLKL and caspase-8 further suppresses IL-1β release and cell death, indicating their cooperative role in IAV-induced inflammatory responses (39) (**Figure 1**). In addition to pyroptosis, apoptosis, and necroptosis, ferroptosis has recently been implicated in influenza virus infection. The HA of PR8 H1N1 induces ferroptosis in the A549 lung epithelial cells, promoting IAV replication. Specifically, the infection leads to increased lipid peroxidation and reactive oxygen species (40), due to intracellular iron accumulation, a hallmark of ferroptosis (41). In mice, IAV-induced ferroptosis contributes to acute lung injury (42). This process is regulated by indoleamine 2,3-dioxygenase 1, which is upregulated by proinflammatory cytokines like interferon-γ (IFN-γ) and promotes ferroptosis in epithelial cells by enhancing oxidative stress and iron accumulation (42, 43) (**Figure 1**).

Similarly, cell death also plays a key role in SARS-CoV-2 pathogenesis. The virus induced necroptosis via Z-RNA/ZBP1 axis in lung epithelial cells (9, 44) and through MLKL activation in monocytes (45). In ACE2-expressing airway cultures, it triggered necroptosis in infected cells and apoptosis in bystander (uninfected) cells, followed by GSDME-mediated pyroptosis at later stages (44). Notably, necroptosis—rather than apoptosis or pyroptosis—has been identified as the primary driver of epithelial barrier disruption, as shown by altered ZO-1 and tubulin expression (46). The necroptosis induced inflammatory cytokines, immune cell infiltration, and lung injury, demonstrated in human lung adenocarcinoma (Calu-3) cells and ZBP1 or RIPK3 knockout models (9). Inflammatory cell death and PANoptosis—induced by ZBP1 activation during SARS-CoV-2 infection—amplified disease severity by contributing to the cytokine storm (47). Among the cytokines elevated during SARS-CoV-2 infection, excessive production of TNF-α and IFN-γ has been shown to drive inflammatory cell death, epithelial injury, and increased mortality due to cytokine shock (48). Beyond the virus itself, anti-SARS-CoV-2 antibodies could also contribute to necroptosis. Immune complexes formed by UV-inactivated SARS-CoV-2 and patient sera—containing anti-SARS-CoV-2 antibodies—have been shown to induce necroptosis in monocytes in a manner dependent on RIPK3, MLKL, and Syk (45). Since monocyte Fcγ receptors recognize IgG complexes and signal through Syk, this process is likely mediated by Fcγ receptor-dependent signaling.

Overall, cell death components such as RIPKs, MLKL, and caspases act in a context-dependent manner, modulating immune responses. During viral infection, RIPK3 functions either as a kinase to induce necroptosis (8, 21) or as a scaffold to promote apoptosis and immune cell recruitment (21, 49). Importantly, as a means to suppress IAV-induced inflammation, Guatam et al. identified a RIPK3 kinase inhibitor, UH15-38, that selectively blocks necroptotic pathways in infected epithelial cells (50). Remarkably, it provided greater protection than RIPK3 knockout by inhibiting necroptosis (RIPK3 kinase activity-dependent), while preserving apoptosis (not kinase-dependent). This shows that selective inhibition of RIPK3 kinase activity effectively alleviates IAV-induced severe tissue damage, providing an example of implicating cell death pathways for therapeutics (50).

### 2.2 Beneficial roles in antiviral immunity

Although excessive activation of cell death and DAMP pathways can be detrimental, their proper activation offers several advantages, including the elimination of infected or damaged cells and the recruitment of innate immune cells. However, the extent of their contribution to antiviral immunity may vary depending on the virus type, viral load, and stage of infection. Excessive activation of the NLRP3 inflammasome contributes to disease pathogenesis; however, its essential role in host immune defense against viral infection has also been demonstrated. Specifically, deletion of *Nlrp3* or *Pycard*—but not *Nlrc4*—in mice increased mortality and impaired viral clearance despite reduced inflammation (51). Moreover, caspase-1-deficient mice were more susceptible to IAV, exhibiting higher mortality and weaker innate immune responses including reduced neutrophils and monocytic dendritic cells (DCs) in BALF (52). While the inflammasome is essential for initiating innate immunity, its role in adaptive responses remains controversial. For example, caspase-1 deletion affected antibody levels during mild PR8 H1N1 infection (10 PFU, 0.4LD_50_) (53), but not during sublethal infection (4000 EID_50_, 0.5LD_50_) in another study (52).

Whereas the NLRP3 inflammasome contributes to host defense through pyroptosis and cytokine release, alternative approaches have emerged that exploit cell death pathways to directly eliminate the virus. That is, one innovative approach selectively induced ferroptosis in viral envelopes while inhibiting it in host cells. Metastable iron sulfides (mFeS) induced ferroptotic death of extracellular PR8 H1N1 virus particles by increasing malondialdehyde and viral lipid peroxidation, reducing infectivity *in vitro*. Furthermore, decocted mFeS inhibited cellular ferroptosis and improved survival in infected mice, highlighting viral ferroptosis as a potential therapeutic target in influenza (54). Additionally, the protective role of cell death and DAMP-associated pathways is particularly evident in herpes simplex virus type 1 (HSV-1) infection, where PANoptosis is triggered through the cooperative actions of AIM2, pyrin, and ZBP1 in macrophages. Remarkably, loss of these PANoptosis components increased mortality during HSV-1 infection, highlighting the antiviral function of PANoptosis in this context (22).

Although cell death pathways and their associated factors support antiviral responses against IAV and HSV-1, their role in SARS-CoV-2 infection appears less protective. In particular, SARS-CoV-2-induced PANoptosis primarily contributes to pathology rather than defense (31). Notably, SARS-CoV-2 has been shown to suppress type I IFN production to evade early immunity (55–57). This immune evasion may lead to immune suppression during the early phase of infection, allowing viral replication and ultimately triggering inflammatory cell death and cytokine storms at later stages. These findings suggest potential benefits of type I IFN-based therapies. However, since IFNAR signaling is linked to ZBP1-driven inflammatory cell death—which may itself contribute to disease pathogenesis (32)—precise regulation of ZBP1-mediated cell death remains a critical challenge in the development of IFN-based treatments (47).

In addition to modulating type I IFN production, IAV and SARS-CoV-2 can actively evade or manipulate cell death-mediated host defense mechanisms. In the case of necroptosis, various viruses deploy their viral proteins to interact with host RIP homotypic interaction motif (RHIM) domain-containing proteins such as RIPK1, TRIF, and ZBP1 to inhibit necroptosis. However, IAV does not have any known inhibitors of caspase-8 or necroptosis. Instead, IAV activates both caspase-8-dependent apoptosis and MLKL-dependent necroptosis during infection, which paradoxically contributes to both viral replication and protective immunity (58). For pyroptosis, the IAV protein NS1 was shown to inhibit NLRP3 inflammasome activation, which may allow the virus to evade the early stages of host defense (59). In contrast, the modulation of pyroptosis by SARS-CoV-2 remains controversial. Some studies have reported that the SARS-CoV-2 nucleocapsid protein suppressed pyroptosis by inhibiting GSDMD cleavage in human monocytes (60). Conversely, other studies have shown that the nucleocapsid protein can interact with NLRP3 and promote inflammasome activation by enhancing the binding between NLRP3 and ASC in A549 and THP-1 *in vitro*. Furthermore, administration of nucleocapsid-expressing adenoviral vectors in mice induced lung injury via NLRP3 inflammasome activation (61). Understanding the *in vivo* consequences of these viral strategies to evade or manipulate cell death pathways will be critical for identifying potential targets for antiviral therapeutics.

## 3 Cell death and DAMP pathways for optimal vaccine responses

Vaccines traditionally used inactivated or attenuated pathogens to induce immune responses. Due to safety concerns, modern vaccines often rely on purified antigens, which tend to be less immunogenic. To enhance their efficacy, adjuvants are commonly used. Because vaccines selectively mimic natural infection, the immunogenicity of certain vaccines critically depends not only on microbial patterns but also on cell death and DAMP pathways (62). Indeed, cell death-driven immunogenicity has been identified in studies of inactivated vaccines and adjuvants, revealing mechanistic links between cell death and activation of innate and adaptive immunity.

Firstly, inactivated influenza virus vaccine has been shown to induce necrosis-like death of lymph node (LN) macrophages (**Table 1**). These macrophages, particularly medullary macrophages rather than subcapsular sinus macrophages, were shown to be key producers of IFN-β following vaccination. IFN-β subsequently promoted IL-1α secretion via DC activation, which was essential for B cell responses in the draining LN (dLN) (18). The role of macrophage cell death was supported by findings showing that macrophage depletion via clodronate liposome treatment reduced LN DC activation in mice. Humoral responses were further confirmed by the reduced numbers of influenza-specific IgG antibody-secreting cells (ASCs) in the LNs of mice deficient in IL-1α or the IL-1 receptor, and conversely, by the increased numbers in mice treated with IL-1α or IFN-β (18). These findings highlight vaccine-induced cell death as an important trigger of immune activation.

**Table 1:** Cell death and DAMP pathways in vaccine responses.

Vaccine/adjuvant type		Cell death/DAMP	Outcomes
Inactivated IAV vaccine		Necrotic cell death (dLN macrophages)	Production of IFN-β by macrophages → IL-1α production → DC activation → Antibody responses (IgG ASC) (18)
Alum		Necrotic cell death (cathepsin B-dependent)	Possibly Th2-associated immune responses (64)
		Neutrophil cell death	NET formation → IgG production (65)
		Uric acid	Inflammatory monocyte recruitment, T cell proliferation (66)
		DNA	Adaptive immune responses (Th2 responses, IgG production, IgE isotype switching) (19)
		NLRP3 inflammasome	Unknown (not required for inducing adaptive immune responses) (72)
SE adjuvants		Cellular internalization of the oil-in-water emulsions → Necroptosis (dLN macrophages)	DC activation → CD8^+^ T cell responses (20)
		Uric acid (dLN)	CD4^+^& CD8^+^ T cell responses, Antibody responses (targeting DCs & B cells) (24)
		ATP (muscle tissues at injection sites)	CD4^+^& CD8^+^ T cell responses, Antibody responses (23)
Saponin-based adjuvant	QS-21 alone	NLRP3 inflammasome (via canonical pathway)	Reduce vaccine effects (78)
	QS-21 (formulated in cholesterol-containing liposomes)	NLRP3 inflammasome (dLN macrophages, via canonical pathway & TLR4 signaling pathway)	CD8^+^ T cell responses (77)
		HMGB1 (dLN)	CD4^+^ T cell responses (77)
		Lysosomal destabilization (cathepsin B-dependent)	DC activation (Syk activation) → CD4^+^ & CD8^+^ T cell responses (but not antibody responses) (80)

DAMP, damage-associated molecular pattern; IAV, influenza A virus; dLN, draining lymph node; IFN-β, interferon-β; IL-1α, interleukin-1α; DC, dendritic cell; Th2, T helper 2 cell; NET, neutrophil extracellular trap; Ig, immunoglobulin; DNA, deoxyribonucleic acid; NLRP3, nucleotide-binding oligomerization domain (NOD)-like receptor (NLR) family pyrin domain-containing; ATP, adenosine triphosphate; CD, cluster of differentiation; HMGB1, high-mobility group protein B1; MPLA, monophosphoryl lipid A.→ means consequent outcomes.

In line with this concept, adjuvants can enhance immunogenicity by engaging cell death pathways. Notable examples include alum, squalene-based emulsions (SE), and saponin-based adjuvants. Alum, a widely used adjuvant, exemplifies how the cell death pathway can initiate immune activation (**Table 1**). While once thought to act primarily through a depot effect at the injection site, this mechanism is now considered to have little relevance to adaptive immunity or antigen presentation by B cells and dendritic cell subsets (63). Instead, alum has been shown to induce necrotic cell death in myeloid leukocytes through cathepsin-dependent lysosomal disruption, highlighting a direct cellular mechanism contributing to its adjuvanticity (64). Moreover, alum has been shown to trigger rapid neutrophil recruitment and neutrophil cell death at the injection site. The subsequent extracellular release of host DNA and peptidylarginine deiminase 4-dependent formation of NETs were critical for the alum’s adjuvant activity (65). As such, alum promoted the release of DAMPs which contributed to its adjuvant activity. Uric acid released could activate DCs, thereby promoting the initiation of adaptive immune responses (66). Similarly, host-derived DNA acted as an endogenous adjuvant by enhancing IgG1 production through IRF3-independent pathways and by inducing Th2 responses associated with IgE isotype switching (19). Beyond inducing cell death and DAMP release, alum activated the NLRP3 inflammasome, leading to the production of IL-1β (67–71). However, the extent to which this contributed directly to adaptive immunity remained uncertain. In fact, CD4^+^ and CD8^+^ T cell, as well as antibody production, were largely unaffected by the depletion of NLRP3, caspase-1, or ASC (72, 73). Together, alum has been shown to enhance immune responses through multiple, complementary mechanisms—including inflammasome-independent necrotic cell death, the release of DAMPs, and subsequent activation of innate and adaptive immunity.

SE adjuvants are oil-in-water emulsions containing squalene oil. SE adjuvants, such as MF59 (Novartis) and AS03 (GSK), are incorporated in protein subunit vaccines for influenza (e.g., Fluad, Pandemrix, Arepanrix) and SARS-CoV-2 (e.g., Covifenz, SKYCovione). The immunogenicity of SE adjuvants has been shown to depend on the oil-in-water emulsion formulation. Individual components had no significant adjuvant effect (74) and the adjuvant required cellular internalization for activity (20, 75). This internalization may have allowed surfactant components to access intracellular targets. Several studies have demonstrated crucial roles of cell death and DAMP-related pathways in SE adjuvant-induced immunogenicity (**Table 1**). First, ATP released from muscle tissue has been shown to act as a DAMP that contributes to SE adjuvant immunogenicity by recruiting immune cells to the injection site and is essential for both T helper cell and antibody responses (23). Next, SE adjuvants have been shown to induce necroptosis of draining lymph node (dLN)-resident macrophages via RIPK3, which is critical for the activation of Batf3^+^ dendritic cells and the induction of CD8^+^ T cell responses (20). Building on this, a follow-up study demonstrated that the DAMP uric acid plays a key role in mediating SE adjuvant immunogenicity. Inhibiting uric acid synthesis during immunization reduced antibody and T cell responses, DC activation, and proinflammatory cytokine production. Conversely, administration of monosodium urate crystals enhanced adaptive immune responses *in vivo* and directly activated DCs and B cells *ex vivo* (24). Together, these studies underscore the importance of cell death-mediated pathways for the optimal immunogenicity of the SE adjuvants.

QS-21, a purified saponin fraction from *Quillaja saponaria* bark, is the well-characterized saponin-based adjuvant. Due to its hemolytic properties (76), it is formulated with cholesterol-containing components, as in AS01 and Matrix-M. AS01, a liposome-based adjuvant composed of QS-21 and monophosphoryl lipid A (MPLA), is used in licensed vaccines, including Shingrix and Mosquirix. Matrix-M, a nanoparticle of QS saponins (including QS-21), cholesterol, and phospholipids, is used in the Novavax SARS-CoV-2 vaccine and the WHO-recommended malaria vaccine R21/Matrix-M. Certain cell death pathways have been demonstrated to contribute to the mechanism of action of saponin-based adjuvants (**Table 1**). First, QS-21 in AS01 activated the NLRP3 inflammasome in dLN CD169^+^ resident macrophages, enhancing CD8^+^ T cell responses in mice (77). Interestingly, QS-21 alone activated the NLRP3 inflammasome but reduced vaccine efficacy *in vivo* (78). This may reflect differing mechanisms: QS-21 alone induces canonical NLRP3 activation, while its combination with MPLA (as in AS01) requires additional TLR4 signaling (78). This dual stimulation likely enables a more coordinated immune response. Indeed, QS-21 and MPLA synergistically enhanced early IFN-γ responses and IgG production, with their complex interplay demonstrated in gene expression profiles at the transcriptional level (79). Second, QS-21 stimulated dLN-resident cells to release the DAMP HMGB1, which partially contributed to CD4^+^ T cell responses (77). Considering both NLRP3 inflammasome activation and DAMP release, LN-resident cells may undergo pyroptosis; however, direct evidence for the cell death in specific cell types remains lacking. Third, QS-21 in cholesterol-containing liposomes was endocytosed in a cholesterol-dependent manner and activated human monocyte-derived DCs. This process was mediated by lysosomal destabilization and subsequent Syk activation in DCs, facilitating both CD4^+^ and CD8^+^ T cell responses. However, its impact on antibody production appeared to be limited (80). Although lysosomal destabilization can lead to necroptosis or apoptosis, the role of these pathways in the QS-21’s adjuvant action remains unclear.

## 4 Concluding remarks

Significant progress has been made in understanding how cell death pathways influence viral infections and vaccinations. In viral infections, early cell death can alert the immune system, promoting the clearance of infected cells and suppressing viral replication. However, in some cases, viruses can evade these defense mechanisms and trigger excessive cell death, leading to elevated inflammation and tissue damage. To our knowledge, RCD in immune cells plays dual roles, whereas RCD in epithelial cells tends to be detrimental. The outcomes may vary depending on factors such as virus type and viral load. Despite advances, key questions remain—such as what determines whether cell death leads to protection or pathology, and which antiviral mechanisms are yet to be discovered.

Building on advances in understanding the underlying mechanisms, various therapeutic approaches have been developed to target cell death pathways in the context of infections caused by IAV or SARS-CoV-2. However, those targeting IAV remain at the preclinical stage. These include UH15-38 (RIPK3-mediated necroptosis inhibitor) (50), MCC950 (NLRP3 inhibitor) (81), and ferrostatin-1 (ferroptosis inhibitor) (42). Currently, several agents for treating severe SARS-CoV-2 infection are either in clinical use, approved, or under clinical investigation, most of which target exaggerated inflammation. These include Anakinra (IL-1 receptor inhibitor, in clinical trials) (82, 83), Tocilizumab and Sarilumab (IL-6 receptor inhibitors, FDA-approved in 2022 and under investigation, respectively) (84), Baricitinib (JAK/STAT inhibitor relevant to IFN-mediated PANoptosis, granted EUA by FDA in 2020), and Dexamethasone (an inhibitor of inflammatory cell death, FDA-approved in 2021). Despite this progress, the development and improvement of antiviral therapeutics that harness cell death pathways for viral infections face several challenges. Cell death pathways, particularly early-phase pyroptosis during IAV infection, promote antiviral immunity by removing infected cells and activating immune cells. Thus, untimely inhibition of cell death may suppress these advantageous responses and impair viral clearance. Additionally, the selective inhibition of a single cell death pathway may trigger compensatory activation of alternative cell death modalities. Therefore, precise and context-dependent modulation of pyroptosis, necroptosis, and apoptosis—particularly at phases when excessive inflammation is detrimental—will be essential for effective treatment. However, achieving such precision and translating it into clinical practice remain significant challenges.

Vaccines activate the innate immunity and elicit antigen-specific T and B cell responses to protect hosts against pathogenic infections. Among various vaccine types, some rely on cell death and DAMP pathways to induce robust immune responses. In such cases, vaccine components can trigger cell death through their chemical or structural properties, such as activating death-inducing complexes or causing lysosomal damage. This, in turn, stimulates innate immune cells, as well as T and B cells, thereby enhancing vaccine efficacy. Although recent advances have highlighted the role of RCD and DAMP pathways in shaping vaccine responses—particularly in the context of adjuvant effects—there remains a lack of evidence regarding their involvement across various vaccine platforms, including mRNA, DNA and viral vector vaccines. As this is an emerging field, further exploration of RCD and DAMP pathways holds significant potential for advancing vaccine development and optimization strategies. To achieve this, scientists will need to elucidate context-dependent mechanisms, consider differential responses under various translational conditions (e.g., subject age, disease status such as immunodeficiency, and variations in routes of administration), and identify appropriate stimulatory agents.

Taken together, given that fine-tuned regulation of cell death pathways can yield different outcomes in vaccination and infection, further understanding of their immunogenic and inflammatory roles will provide important insights for vaccine development and the optimization of antiviral therapeutics.
